# Comparative genome analysis of *Bacillus thuringiensis* strain HD521 and HS18-1

**DOI:** 10.1038/s41598-021-96133-w

**Published:** 2021-08-16

**Authors:** Hongwei Sun, Xing Xiang, Qiao Li, Hui Lin, Xiaolin Wang, Jie Sun, Long Luo, Aiping Zheng

**Affiliations:** 1 College of Agronomy, Sichuan Agricultural University, Wenjiang, 611130 China; 2 Wenshan Academy of Agricultural Sciences, Wenshan, 663000 China; 3 College of Agronomy, Shihezi University, Shihezi, 832003 China

**Keywords:** Genetics, Genome

## Abstract

*Bacillus thuringiensis* (*Bt*) is an important biological insecticide used to management of different agricultural pests by producing toxic parasporal crystals proteins. Strain HD521 has an antagonistic effect against *Rhizoctonia solani* AG1IA, the causal agent of rice sheath blight. This strain with three *cry7* genes can the formation of bipyramidal parasporal crystals (BPCs). BPCs are used for insecticidal activities against *Henosepilachna vigintioctomaculata* larva (Coleoptera). Strain HS18-1 contains different types of BPCs encoding genes and has effective toxicity for Lepidoptera and Diptera insects. Here we report the whole genome sequencing and assembly of HD521 and HS18-1 strains and analyzed the genome constitution covering virulence factors, types of plasmid, insertion sequences, and prophage sequences. The results showed that the genome of strain HD521 contains a circular chromosome and six circular plasmids, encoding eight types of virulence protein factors [Immune Inhibitor A, Hemolytic Enterotoxin, S-layer protein, Phospholipase C, Zwittermicin A-resistance protein, Metalloprotease, Chitinase, and *N*-acyl homoserine lactonase (AiiA)], four families of insertion sequence, and comprises six pro-phage sequences. The genome of strain HS18-1 contains one circular chromosome and nine circular plasmids, encoding five types of virulence protein factors [Hemolytic Enterotoxin, S-layer protein, Phospholipase C, Chitinase, and *N*-acyl homoserine lactonase (AiiA)] and four families of insertion sequence, and comprises of three pro-phage sequences. The obtained results will contribute to deeply understand the *B. thuringiensis* strain HD521 and HS18-1 at the genomic level.

## Introduction

*Bacillus thuringiensis* (*Bt*) is a ubiquitous, Gram-positive, spore-forming bacterium^1^. The strains of this species are used as a successful biopesticide in many countries. It can produce insecticidal parasporal crystal proteins^2,3^. These crystal proteins are known as δ-endotoxins, which are specifically toxic to different pests, including species of the Lepidoptera, Coleoptera, Diptera, Hymenoptera, and Homoptera, as well as some nematodes^1,4^. Due to the toxins specific insecticidal activities, they are non-toxic to humans, therefore which were widely used to control insects in agriculture^5,6^. A previous study showed that Bt could also produce some antibiotics, such as Zwittermycin A, to enhance its insecticidal toxins and inhibit pathogenic bacteria^7–9^. The complete antibiotic biosynthesis gene cluster was first identified in the strain *B. cereus* UW85^10^. *B. thuringiensis* can also produce some virulence factors when insects are infected, such as enhances and collagenases^11,12^. Plasmid often encodes many virulence genes play significant roles in pathogenesis in these bacteria. Virulence genes are located on the plasmids usually give rise to different phenotypes and pathologies^13^. The availability of the genome sequences of *B. cereus* group members such as *B. anthracis* A2012^14^, *B. cereus* ATCC 14579^15^, and *B. anthracis* Ames^16^, play an important role in supporting the identification of unique metabolism, comparative physiology, sporulation, and virulence.

To date, many countries were interesting to dig the resources of *Bt* strains, and thousands of *Bt* strains were isolated. But the genomic informations of most strains are not complete, especially those highly toxic strains used for pest control since the last century. Although 42 *B. thuringiensis* strains have been sequenced, gapless chromosomes and plasmids have only been obtained from 15 strains’ (http://www.ncbi.nlm.nih.gov/genome/genomes/486)^17^ In this study, based on the previous research, in order to have a deeper understanding of the structure, function, regulation mechanism and evolutionary laws of strains HD521 and HS18-1, and to provide a scientific basis for inhibiting the pathogenicity of pathogenic bacteria and preventing insect pests. Here, we introduced the genome information of the two strains in detail.

## Materials and methods

### Materials

The strain HD521 belongs to the Indiana subspecies (Indiana) and was obtained from Bacillus Genetic Stock Center (BGSC). Strain HS18-1 was isolated from the Sichuan Basin of China and was stored in our laboratory. Genomic extract reagent (QIAGEN Genomic-tip 500/G) was purchased from QIAGENgs, The cloning vector Trans1-T1 and *E. coli* DH5α were purchased from the full-scale gold company.

## Methods

### Sequencing and assembly of strain HD521

The mixture of total DNA and plasmid of strain HD521 is randomly interrupted by using Ultrasonic Disruptor (method: ultrasound for 30 s, with an interval of 30 s, 10 cycles is 1 time; take out the sample, vortex and mix, then put it back into ultrasonic breaker to continue ultrasonic interruption. The whole process need three times). Then construct a sequencing library for the interrupted total DNA according to Illumina’s paired-end library construction kit was done. Finally, Sequencing using Illumina HiSeq 2000 platform at Beijing Genomics Institute (BGI; Shenzhen, China) (BGI) was completed. The sequencing structure was de nove assembled by using software Velvet 1.2.10^18^. Simultaneously, paired-end reads sequencing are anchored to the assembled fragments by using software Burrows–Wheeler Aligner’s Smith–Waterman Alignment to verify the accuracy of sequence assembly^19^.

### Sequencing and assembly of strain HS18-1

The genome sequencing strategy of strain HS18-1 is the second-generation sequencing technology combined with the third-generation sequencing technology-single molecular, real time, circular consensus sequencing (SMRT), Similar to strain HD521, when sequencing, the Hiseq 2000 platform was used, a pair-end library was constructed, and insert sequence length was two kbp and 200 bp. A library with an insert length of 10 kbp was constructed in single-molecule real-time sequencing, according to the SMRT sequencing library construction instructions. The data obtained by HiSeq 2000 sequencing platform was assembled using the software Velvet, 1.2.10. The obtained sequence is used to assemble and correct the accuracy of some sequences in the third-generation sequencing^20^, the assembly of the third-generation sequencing data using SMRT analysis assembly software Velvet, 2.3.0^21^.

### Assembly and result verification of complete genome-wide maps

The results of cloning and sequencing are compared with the border sequences on Scaffolds, more than 99% completely matches sequence in the border sequences been known as a gap closing sequence, so this corresponding fragment can connect two different scaffolds, Some sequencing results cannot completely match with the boundary sequence of two Scaffolds, but it can be matched with the boundary sequence of one of the Scaffolds. In this case, we consider that the sequence can effectively extend the matching Scaffold. Then to design primers, amplify, clone, and compare from newly extended sequences until the two Scaffolds are spliced together.

In the process of sequence splicing, if there is some genomic repetitive sequences and regions with low base reliability, their corresponding primers are designed, to amplify, clone, and sequencing verification. In this way, the error rate of the genome is lower than one bp/10 kb, thereby improving the accuracy of genome splicing and assembly.

### Gene annotation of genomes and plasmids, and prediction of open reading frames

The Open Reading Frame (ORF) of whole genome of HD521 and HS18-1 was predicted by using GeneMarks software with default parameters^22^. The protein coding sequences of whole genome were predicted by applying Glimmer 3.02^23^, rRNA, tRNA and sRNA were predicted by rRNAmmer, tRNAscan and Rfam, respectively^24–26^. The genes of signal peptides and transmembrane helices were predicted by SinnalP 3.0 and TMHMM 2.0^27,28^.

### Circular genome graph of strain HD521 and HS18-1

The circle graph of genome and plasmid of strain HD521 and HS18-1 was drew by using Circos software (Biomarker Biotechnology Co., Ltd; Beijing, China), The circle graph indicates the scale ruler, the forward strand and reverse strand of DNA, and the GC content of the genome; GC skew; classification of different genes.

### Analysis of genome insertion sequence of strain HD521 and HS18-1

According to the genome data of HD521 and HS18-1, Insertion sequence (IS) of genomic and plasmid sequences were aligned by using the ISsaga program in IS FINDER, and the distribution of IS sequences was analyzed (http://issaga.biotoul.fr/issaga_index.php). After confirmation of the transposase gene, the selected sequence at the upper and lower 300 bp nucleotide sequence of the transposase gene was aligned for the analysis of IS sequence structure. IR region of IS sequence and DR region at the both ends of IR region was found. For some IS sequences, if IR region cannot be confirmed, the search range of upstream and downstream sequences at the transposase gene was enlarged to find and confirm the DR region.

### Sequence analysis of HD521 and HS18-1 genome prophage

The distribution of whole genome sequence and lysogenic phage sequences in plasmids of HD521 and HS18-1 were analyzed by using PHAST (http://phast.wishartlab.com/index.html)^29,30^.

### Virulence factors analysis of HD521 and HS18-1 genome

Virulence factor gene and protein sequence information in Bacillus cereus group (*B. cereus*, *B. anthrax* and *B. thuringiensis*) were collected, and a virulence factor gene set (protein sequence set) is constructed, and then Homology comparison the annotation results of HD521 and HS18-1 genomes and plasmids with the gene set, then get a gene set of predicting virulence genes and their sequences were analyzed by using BLAST software, Finally, the virulence factor sequence information of HD521 and HS18-1 were obtained.

## Results and analysis

### Features of strain HD521 and HS18-1

*Bt* strain HD521 was first isolated from soil sample of the United States^31^. It was obtained from Bacillus Genetic Stock Center (BGSC). Strain HD521, like the majority of the *Bt* strains, cells are Gram-positive and rod-shaped^1^. It exhibits maroon colonies and produces bipyramidal parasporal crystals (BPCs) during the stationary phase of its growth cycle. But, the difference is that its colonies can produce brown–red pigments that turn the entire colony into brownish. SDS-PAGE analysis of spores and crystals mixtures showed the strain HD521 expression of a major protein band of 130 kDa, which is consistent with the following analysis of its parasporal crystal gene^32^. However, strain HS18-1 was isolated from the Sichuan basin of China, and it has typical toxicity against Lepidoptera and Diptera^33^. It can produce spherical parasporal crystals during the stationary phase of its growth cycle. SDS-PAGE analysis of spores and crystals mixtures showed HS18-1 expression of two major protein band of 130 kDa and 75 kDa^34^. By identifying insecticidal gene, it indicated that strain HS18-1 contains very rich cry-type insecticidal crystal protein genes, including cry4Cb1, cry30Ga1, cry30Ea1, cry56Aa3, cry50Aa, cry69Ab1, cry70Aa, cry71Aa, and cry72Aa^33,34^.

### Genomic composition of strain HD521 and HS18-1

**Figure 1:**
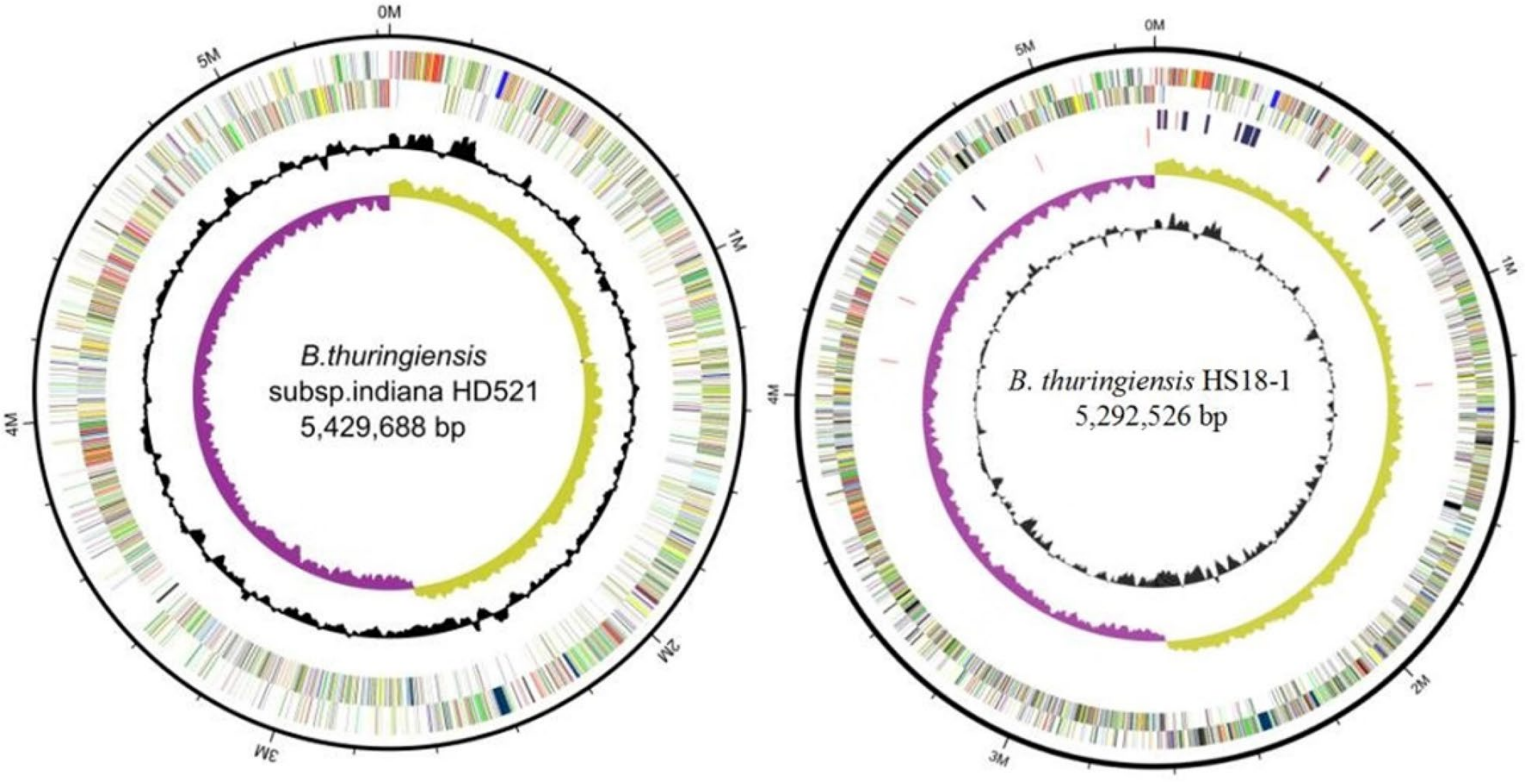
Circular pictorial representation of chromosome of both *Bt* strains; (**A**) *Bt* strain HD521 and (**B**) *Bt* strain HS18-1. From outside to center: the first circle, the forward strand of DNA; the second circle, the reverse strand of DNA; the third circle GC content (black), the fifth circle GC skew.

The genome of HD521 consisted of seven replicons: a circular chromosome (Fig. 1) and 6 circular plasmids. The GC content of the circular chromosome with a length of 5,429,688 bp is 35.28%. It included a predicted 5538 genes and 138 are RNA genes. Total of these 5400 genes with a collective length of 4,544,493 bp, are protein-encoding genes. 6 of the plasmids are named pBTHD521-1, pBTHD521-2, pBTHD521-3, pBTHD521-4, pBTHD521-5, and pBTHD521-6. The GC content of the six plasmids ranged from 29.45 to 35.91% and contained a total of 772 predicted genes. However, the genome of HS18-1 consists of 10 replicons with one circular chromosome (Fig. 1) and 9 circular plasmids. The gapless circular chromosome with a length of 5,292,526 bp (35.43% GC content) contains 5382 genes, 148 of total are RNA genes, and total of these, 5234 are protein-encoding genes. 9 of the plasmids are named pHS18-1, pHS18-2, pHS18-3, pHS18-4, pHS18-5, pHS18-6, pHS18-7, pHS18-8, and pHS18-9. The GC content of 9 plasmids is ranged from 28.49 to 37.07% and consist total 892 predicted genes (Table 1).

**Table 1:** Genomic composition of *Bacillus thuringiensis* strain HD521 and HS18-1.

Feathers	Length(bp)	HD521				Feathers	Length (bp)	HS18-1			
		G + C content (%)	Coding genes	rRNA	tRNA			G + C content (%)	Coding genes	rRNA	tRNA
Chromosome	5,429,688	35.28	5538	31	107	Chromosome	5,292,526	35.43	5382	42	106
Plasmid pBTHD521-1	7042	11	29.45	0	0	Plasmid pHS18-1	509,170	32.71	417	0	0
Plasmid pBTHD521-2	49,838	70	35.91	0	0	Plasmid pHS18-2	337,579	33.25	357	0	0
Plasmid pBTHD521-3	71,771	89	34.37	0	0	Plasmid pHS18-3	92,085	31.06	91	0	0
Plasmid pBTHD521-4	71,646	103	29.79	0	0	Plasmid pHS18-4	94,695	33.54	79	0	0
Plasmid pBTHD521-5	253,580	243	33.08	0	0	Plasmid pHS18-5	42,726	42,726	59	0	0
Plasmid pBTHD521-6	314,883	256	31.99	0	0	Plasmid pHS18-6	14,336	36.17	9	0	0
						Plasmid pHS18-7	4669	37.07	7	0	0
						Plasmid pHS18-8	8287	28.49	6	0	0
						Plasmid pHS18-9	7386	32.29	4	0	0

Although both the HD521 and HS18-1 genomes carry plasmids, but the HS18-1 have more plasmids than the HD521, and reached 9. The largest plasmid pHS18-1 is 509,170 bp, the smallest plasmid pHS18-9 is 7386 bp.

### Virulence factors

The insecticidal active ingredients of strains HD521 and HS18-1 are mainly encoded insecticidal crystal proteins on the plasmid. In addition, the chromosomes also encode a large number of insecticidal active ingredients (Table 2), and their insecticidal mechanisms are also different. Moreover, there is synergistic effect between the insecticidal active ingredients.

**Table 2:** Virulence factors of *Bacillus thuringiensis* strain HD521 and HS18-1.

Virulence factor	HD521		Virulence factor	HS18-1		
	Annotation Number	Gene function		Annotation Number	Gene function	
Immune inhibitor	NF53_1173	Immune inhibitor A				
	NF53_2862	Immune inhibitor A				
	NF53_2863	Immune inhibitor A				
Hemolytic enterotoxin	NF53_1020	Trifolitoxin immunity domain protein	Hemolytic enterotoxin	AC241_11040	Hemolysin D	
	NF53_1002	Gamma-hemolysin component B		AC241_11455	Hemolysin BL lytic component L2	
	NF53_1695	Hemolytic enterotoxin		AC241_17290	Hemolysin II	
	NF53_1696	Non-hemolytic enterotoxinlytic component L1		AC241_27105	Hemolysin	
	NF53_2062	Hemolysin-3		AC241_29375	Hemolysin BL lytic component L2	
	NF53_3001	Hemolysin BL-binding componen		AC241_29380	Hemolysin BL lytic component L1	
	NF53_3002	Hemolysin BL-binding component		AC241_29385	Hemolysin	
	NF53_3003	Tripartite hemolysin BL component L1		AC241_30075	Hemolysin D	
	NF53_3004	Hemolysin BL lytic component L2				
	NF53_3004	Hemolysin-3				
S-laryer protein	NF53_0786	S-layer domain protein	S-laryer protein	AC241_02460	S-layer protein	
	NF53_0890	S-layer Protein/peptidoglycanendo-beta-*N*-acetylglucosaminidase		AC241_04995	S-layer protein	
	NF53_0997	S-layer protein		AC241_05720	S-layer protein	
	NF53_1016	S-layer protein		AC241_05725	S-layer protein	
	NF53_1803	S-layer Protein/peptidoglycanendo-beta-*N*-acetylglucosaminidase		AC241_30720	S-layer protein	
Phospholipase C	NF53_0580	Phospholipase C	Phospholipase C	AC241_03515	Phospholipase C	
	NF53_2241	Phosphoesterase		AC241_03520	Phospholipase C	
	NF53_2399	Patatin phospholipase		AC241_10060	Phospholipase	
	NF53_4723	Phospholipase YtpA		AC241_23600	Phospholipase	
	NF53_0581	Sphingomyelinase C				
		1-phosphatidylinositol phosphodiesterase		AC241_31980	Phospholipase C	
Zwittermicin A-resistance protein	NF NF53_370153_2753	Zwittermicin A-resistance protein				
	NF53_3078	Zwittermicin A resistance protein zmaR				
Metalloprotease	NF53_1014	Metalloprotease				
	NF53_1365	Metalloprotease				
	NF53_2033	Neutral metalloprotease				
Chitinase	NF53_0359	Chitinase A1	Chitinase	AC241_02250	Chitinase	
	NF53_3663	Chitinase D		AC241_18365	Chitinase	
AiiA	NF_3317	*N*-Acyl homoserine lactonase, AiiA	AiiA	AC241_03670	*N*-Acyl homoserine lactonase, AiiA	
				AC241_06965	*N*-Acyl homoserine lactonase	
				AC241_16885	*N*-Acyl homoserine lactonase	
				AC241_24135	*N*-Acyl homoserine lactonase	
				AC241_24135	*N*-Acyl homoserine lactonase	

Strain HD521 comprises a plethora of virulence factors such as Immune Inhibitor A, Hemolytic Enterotoxin, S-laryer protein, Phospholipase C, Zwittermicin A-resistance protein, Metalloprotease, Chitinase, and *N*-acyl homoserine lactonase (AiiA). Immune Inhibitor A is a metallo-enzyme, it have three copies on the chromosome of strain HD521, which able to enhance toxicity to insects by inhibiting insect immune factors and hydrolyzing some antibacterial proteins in insects. The chromosome of strain HD521 encodes 9 enterotoxin genes, it is a virulence factor contained in *Bacillus cereus* which causes vomiting and diarrhea in humans. However, we conclude that strain HD521 may have hemolytic properties due to 3 subunit genes, Gamma-hemolysin component B, Tripartite hemolysin BL component L1, and Hemolysin BL lytic component L2, encoded in chromosome of strain HD521.

The S-layer protein forms an ordered crystal array structure on the surface of pathogenic bacteria that maintains cell morphology and cell integrity, so it belongs to a class of surface proteins and is widely distributed in *Bacillus* species. The S-layer protein of *Bt* have synergistic effects on insecticidal crystal proteins. Phospholipase C can hydrolyze phosphatidyl alcohol and phosphatidyl choline, which can also cause certain damage to the intestinal tract of insects and promote the activity of insecticidal crystal proteins in a certain extent^35^. Zwittermicin is a new broad-spectrum antibiotic that can inhibit the growth of a variety of microorganisms, especially oomycetes and their related bacteria. But the chromosome of *Bt* HD521 contains two resistant genes for Zwittermicin, Zwittermicin A-resistance protein and Zwittermicin A resistance protein zmaR. Therefore, we conclude that *Bt* HD521 has certain resistance to Zwittermicin. Chitin is also known as shell polysaccharide, which is widely found in the shells of insects, the shells of crustaceans and the cell walls of fungi, and acts as a support skeleton to protect itself. Meanwhile, Chitinase is an enzyme that can hydrolyzes chitin, its main role is to has a synergistic effect on pesticides. Because the chitin is one of the main component of the insect midgut peritrophic membrane, the peritrophic membrane is the barrier of insects against bacteria and viruses, when the peritrophic membrane is destroyed by chitinase, the activity of insecticidal protein ultimately increases^36–38^. The strain HD521 chromosome encodes two types of chitinases, chitinase A and chitinase D, which can hydrolyze the outer wall of insects and cause insect death. *N*-Acyl homoserine lactonase (AiiA) is an enzyme that can degrade *N*-acyl homoserine lactones (AHLs) and is a signal molecule of bacteria, which acts as a signaling molecule of the gram-negative bacterial quorum sensing system and participates in the expression regulation of the pathogenic genes^39^. The AiiA gene-expressing protein contributes to the degradation of AHL molecules, it can reduce the concentration of AHLs by hydrolyzing the lactone bond of AHLs that declines the harm caused by pathogens. Previous studies have shown that AiiA has the effect of enhancing the resistance of Zwittermicin to soft rot^40^. Therefore, the AiiA gene of strain HD521 may have a synergistic effect to *Rhizoctonia solani* AG 1 IB. The chromosome of strain HS18-1 encodes five virulence factors such as Hemolytic Enterotoxin, S-laryer protein, Phospholipase C, Chitinase, and *N*-acyl homoserine lactonase (AiiA). But strain HS18-1 chromosome have not subunit B which is necessary for hemolytic in enterotoxin. It contains subunit L1 and L2. The chromosome of strain HS18-1 encodes 8 hemolytic enterotoxin, 5 S-laryer proteins, 5 phospholipases, 2 chitinases, and 5*N*-acyl homoserine lactonases (AiiA). In comparison of HS18-1 with HD521, the strain HS18-1 does not encode Immune Inhibitor A, Zwittermicin A-resistance protein and metalloprotease.

### Plasmid analysis

**Figure 2:**
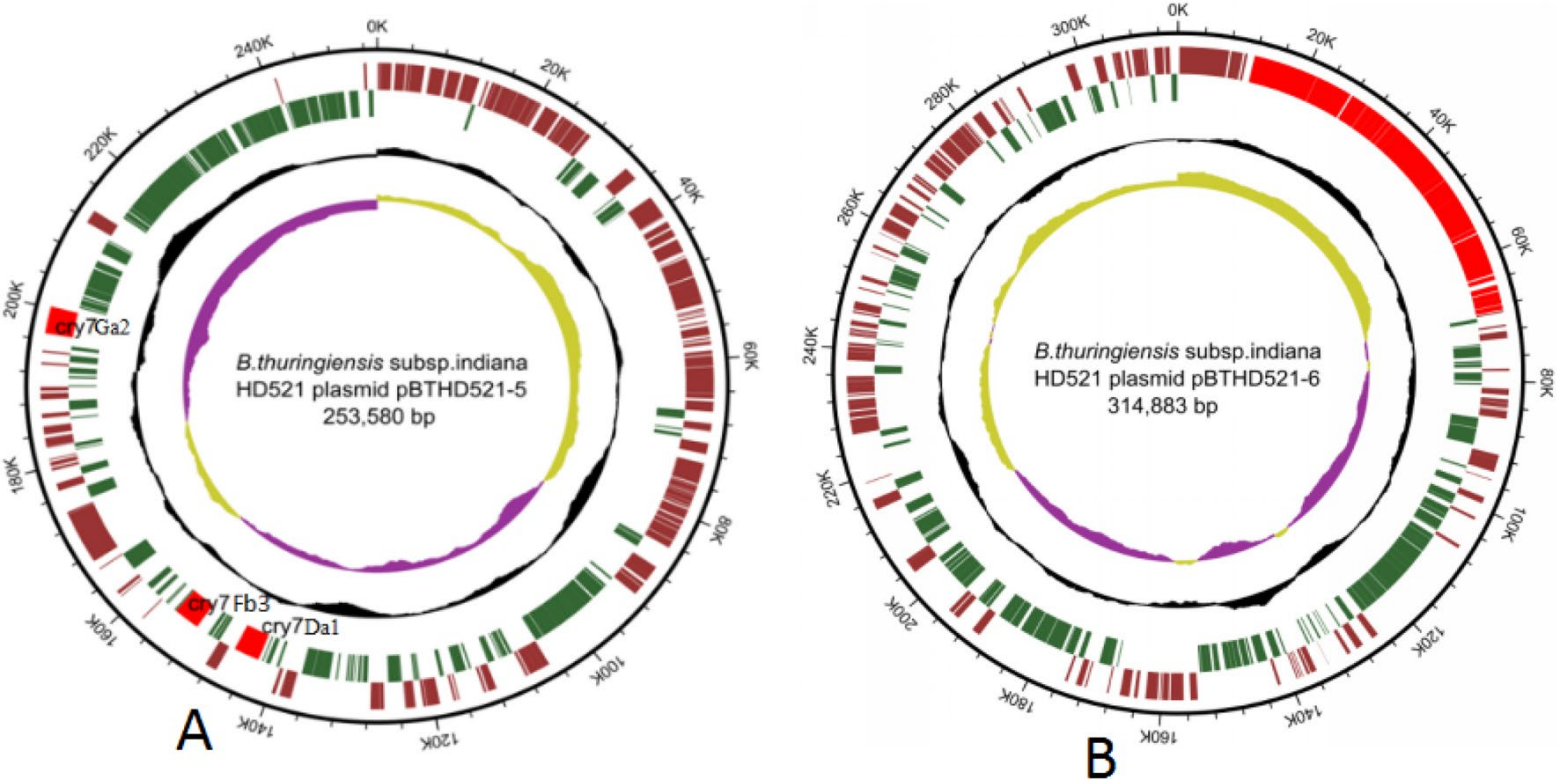
Circular representation of plasmids pBTHD521-5 and pBTHD521-6. (**A**) Circular representation of plasmid pBTHD521-5 displaying relevant genome features. (**B**) Circular representation of plasmidpBTHD521-6 displaying relevant genome features.

*Bt* strain HD521 contains 6 plasmids and codes a total of 772 predicted genes, the smallest plasmid is pBTHD521-1with a length of 7042 bp and encodes 11 functional genes, the largest plasmid is pBTHD521-6 with a length of 314,883 bp and encodes 256 functional genes. Some of them, pBTHD521-5 and pBTHD521-6, are used as plasmid which contain the insecticidal crystal protein. However, pBTHD521-1, pBTHD521-2, pBTHD521-3, and pBTHD521-4 are used as plasmid without any insecticidal crystal protein. The whole length of plasmid pBTHD521-5 is 253,580 bp and encodes three cry7 genes named as cry7Da1, cry7Ga2 and cry7Fb3 (Fig. 2A), the gene cry7Ga2 located on sense strand while gene cry7Fb3 and cry7Da1 located on antisense strand.

The IS6 family of insertion sequence located on the downstream sequence of gene cry7Fb3 and cry7Da1, and the IS231B family of insertion sequence located on the upstream sequence of gene cry7Ga2. Plasmid pBTHD521-5 also encodes the plasmid replication protein RepX and conjugal transfer protein TraG that means the replication mode of this plasmid is bidirectional replication and can transfer between different strains. The whole length of plasmid pBTHD521-6 is 314,883 bp, encoding the ZwA virulence factor and consisted by 22 ZwA biosynthesis relevant genes that are zmaA, zmaB, zmaC, zmaD, zmaE, zmaR, zmaF, zmaG, zmaH, zmaI, zmaJ, zmak, zmaL, zmaM, zmaN, zmaO, zmaP, zmaQ, zmaS, zmaT, zmaU, and zmaV and its through NRPS and PKS biosynthesis pathway to synthesis (Fig. 2B).

**Figure 3:**
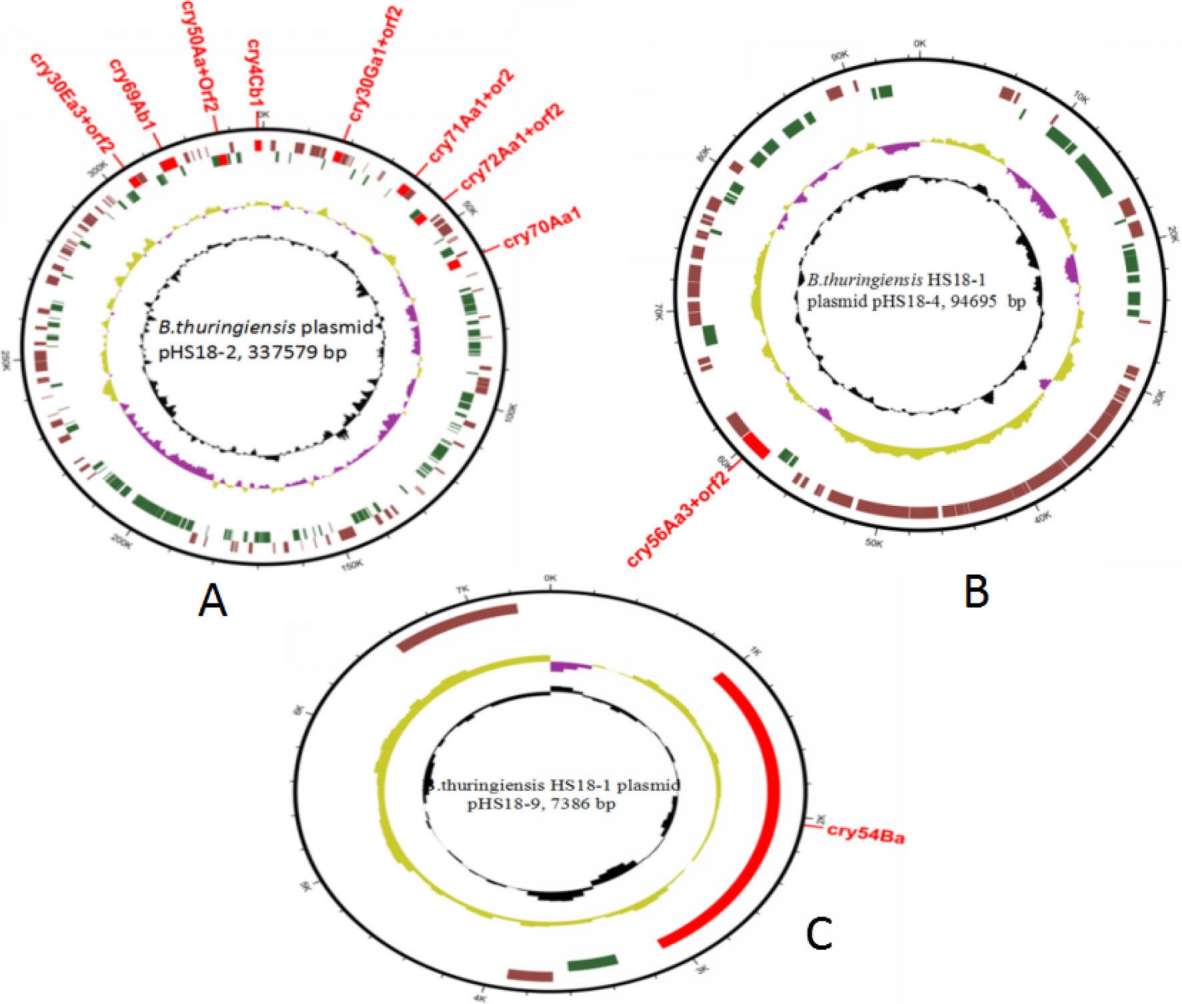
Circular representation of plasmids pHS18-2, pHS18-4 and pHS18-9. (**A**) Circular representation of plasmid pHS18-2 displaying relevant genome features. (**B**) Circular representation of plasmid pHS18-4 displaying relevant genome features. (**C**) Circular representation of plasmid pHS18-9 displaying relevant genome features.

*Bt* strain HS18-1 contains nine plasmids and codes a total of 892 predicted genes, the smallest plasmid is pHS18-9 with a length of 7386 bp that encodes 4 functional genes, and the largest plasmid is pHS18-1 with a length of 509,170 bp that encodes 417 functional genes. Some of them, pHS18-2, pHS18-4 and pHS18-9 are used as plasmids which contain the insecticidal crystal proteins. However, pHS18-1, pHS18-3, pHS18-5, pHS18-6, pHS18-7, and pHS18-8 are the plasmids without any insecticidal crystal protein. Plasmid pHS18-2 encodes eight insecticidal genes e.g.cry30Ea3 + orf2, cry50Aa1 + orf2, cry30Ga1 + orf2, cry71Aa1 + orf2, cry72Aa1 + orf2, cry70Aa1, cry60Ab1, and cry4Cb1 (Fig. 3A). Plasmid pHS18-4 encodes insecticidal gene cry56Aa3 + orf2 (Fig. 3B). Plasmid pHS18-9 encodes insecticidal gene cry54Ba (Fig. 3C).

The insecticidal genes encoded by HS18-1 are like the genes encoded by HD521, beside these genes there are some mobile elements such as IS4 family of insertion sequence located in the downstream sequence of cry30Ga1 + orf2, IS4 sequence located in the in the upstream sequence of cry71Aa1 + orf2, Tn3 and IS231C located in the upstream and downstream sequences of cry30Ea1 + orf2, respectively. And IS231C in the upstream and downstream sequence of cry30Ea1 + orf2, IS231 sequence located in the downstream sequence of cry4Cb1. It reveals that the cry genes combined with mobile elements as genomic island in the genome.

### Insertion sequence analysis

Insertion sequence (IS) is a movable element that causes genomic plasticity and its main feature is the transposition between different sites within the genome. A basic IS element includes a site-specific recombinase (transposase) and flanking repetitive DNA sequences^41^. Different IS sequence elements have great difference in transposition mechanisms and target specific sites, such as IS7 and IS30^42,43^. By comparing ISin the genome of both strains, HD521 and HS18-1 (Table 3), we found that the family and number of IS have significant difference in the distribution of the genome. Among them, the number of distribution of strain HS18-1 is the largest, and the IS family is the most abundant.

**Table 3:** The varieties and quantities of IS in HD521 and HS18-1 genome.

IS sequence family	HD521		IS sequence family	HS18-1	
	Characterization^a^	Start and end position		Characterization^a^	Start and end position
IS*200*_IS*605*_ssgr_IS*1341*	1/0/0/0	3,517,174–3,515,052	IS*200*_IS*605*_ssgr_IS*1314*	1/0/0/1	1,950,239–1,953,704
					4,239,014–4,235,659
IS*6*	1/0/0/0	3,501,376–3,503,581	IS*3*_ssgr_IS*150*	35/1/0/0	1,908,915–1,912,268
					1,024,093–1,020,937
IS*607*	1/0/0/0	332,416–335,545	IS*607*	2/0/0/0	1,222,989–1,226,127
IS*110*	1/0/0/0	3196134–3192899	IS*110*	0/2/0/0	5,004,244–5,007,064
					5,004,244–5,007,064

^a^Complete sequence/partial sequence/pseudogenes/unknown sequence.

IS of four families were found in the genome of HD521 by IS sequence analysis. We found that IS family IS6 and IS110 have one copy, respectively. But the insert sequence of IS200_IS605_ssgr_IS1341 family has two copies (Table 3). Bt HD521 contains 6 plasmids and 4 types of IS sequences. Among them, the plasmids pBTHD521-1, pBTHD521-2, pBTHD521-3 and pBTHD521-4 did not contain any IS and these are small plasmids with length of 7 kbp, 50 kbp, 71 kbp and 71 kbp, respectively. The plasmid-encoded transposase gene analysis revealed that pBTHD521-5 and pBTHD521-6 contain 4 and 3 IS families, respectively. Their IS are mainly focused in the IS200_IS605_ssgr_IS1314, IS6, IS4_ssgr_IS231, and IS200_IS605 families (Table 4). Through the distribution of the IS on the plasmids, we can perceive that the IS of *Bt* is often found in some larger plasmids, so the evolution rate of the endogenous large plasmid of *Bt* is larger than that of the endogenous small plasmid. In the genome and plasmid of HD521, we found that there are 4 IS on the genome and 17 IS on the plasmids. The family of IS shared by the plasmid and genome are IS200_IS605_ssgr_IS1341 and IS6. Besides, the genome also contains two specific families that are IS607 and IS110 and plasmid contains a specific IS231 family along with two subfamilies, IS231B and IS231E. This suggests the indication of the rate of plasmid evolution is faster than the evolution of the genome.

**Table 4:** The total varieties and quantities of IS sequences in HD521 plasmids.

Plasmid	IS sequence family	Characterization^a^	Start and end position (bp)
pBTHD521-1			
pBTHD521-2			
pBTHD521-3			
pBTHD521-4			
pBTHD521-5	IS*200*_IS*605*_ssgr_IS*1341*	3/0/0/0	181570_184684
			69596_72947
			191964_189733
	IS*6*	2/0/0/2	143434_140984
			156586_158982
			152421_150180
			201548_199369
	IS*4*_ssgr_IS*231*	2/0/0/2	145896_149385
			189234_185802
	IS*200*_IS*605*	2/1/0/0	117482_113986
pBTHD521-6	IS*200*_IS*605*_ssgr_IS*1341*	2/0/0/0	203973_200852
	IS*4*_ssgr_IS*231*	5/0/0/0	310195_314508
			238504_235158
			83240_86681
			271106_274295
			267744_269823
	IS*200*_IS*605*	1/0/0/0	194062_192074

^a^Complete sequence/partial sequence/pseudogenes/unknown sequence.

By analysing the HD521 whole-genome annotation results and ISFINDER data, we found a complete IS607 family IS in the HD521 genome. It does not contain an inverted repeat region (IR), but it encodes two ORFs; one encodes a 1131 bp transposase gene, and the other encodes a Haloacid Dehalogenase which plays a crucial role to carry out dehalogenation, phosphoryl transfer and hydrolysis of phosphate salts by forming covalent enzymes.

Meanwhile, we also found a complete IS110 family of IS in the HD521 genome, it also does not contain an IR region with similar to IS family IS200_605, IS607. But the difference is, IS100 family contains a conserved amino-terminal region of pilin gene inverting protein (PIVML). Through analysis we found that IS*110* family includes two ORF sequences, one ORF encodes a 573 bp DNA dissociation enzyme gene and it is a specific DNA recombination site; its N terminus has a DNA activation site while C-terminal can form a helical loop spiral structure. The other one encodes a 1236 bp DNA transposase gene of DEDD_Tnp_IS110 family specific conserved site, which has a great significance to the efficient transposition of DNA.

The first type of IS, IS200_IS605_ssgr_IS1341 on plasmid pBTHD521-5 has 3 copies, which have an ORFB structure similar to IS family of IS110, the C terminus of ORFB has four typical cysteine residues with an ability to combine with zinc. Therefore, the C terminus is mainly used as a DNA binding site in transposition process^44^. By comparing the upstream and downstream genes in IS, we found that the first IS1314 was inserted into C1qtnf9 (C1q and tumor necrosis factor related protein 9) protein of house mouse. However, the 3′ end of the IS1314 sequence can form a complete ORF with the downstream C1qtnf9, thus the insertion of IS1314 sequence brings an exogenous gene to the HD521 genome. But, the insertion of the second IS did not result in a change of functional gene and did not carry the insertion of a new heterologous gene.

According to the transposase gene and ISsaga results, we found that plasmid pBTHD521-6 contains 2 copies of the IS IS200_IS605_ssgr_IS1341 family, 1 copy of the IS IS200_IS605 family, and 5 copies of the IS IS4_ssgr_IS231 family. At the same time, IS4_ssgr_IS231 family also includes subfamily insertion sequences IS231B (238,504–235,158 bp) and IS231E (267,744–269,823 bp), these subfamily insertion sequences generally have a transposase gene which contains ORF containing Integrase binding domain, Helix-turn-helix (HTH) DNA binding site, and DDE structure. The DDE structure has three carboxylic acid residues, and they can combine with metal ions which participate in catalytic DNA cutting to catalytic DNA cleavage, and to transcript regulator. IS231 IS family also contains some enzymes which participate in the metabolism of amino acid and nucleic acid, such as proline dehydrogenase and ribose triphosphate deoxyribonucleoside reductase.

The HS18-1 genome contains 42 copies of the transposase gene and IS family IS3_ssgr_IS150 contains 36 copies, IS200_IS605_ssgr_IS1314, IS607 and IS110 have two copies, respectively (Table 3). However, the transposase gene of IS607 family contains a helix-trans-helical DNA domain at the N terminus and four conserved cysteine residues at the C terminus, and IS110 family has a typical DEDD structure. The IS150 of IS3 family is similar to IS607, also has a helix-trans-helical DNA domain at the N terminus, but contains an integrase core binding domain at its C terminus. The pHS18-2 plasmid encodes 62 transposase genes and contains eight IS families (Table 5). IS family IS200_IS605_ssgr_IS1341 contains a zinc finger structure that combines with DNA at the C-terminal of the transposon, its transposase gene has only 82% homology with other by comparison in NCBI database, which enables to perceive that it might be a new type of IS. IS family IS3_ssgr_IS3 has only 84% homology with the transposase gene of *Bacillus cereus*, its DNA binding domain is a helix-turn-helix structure, and it contains a DNA integrase gene, thus it may also be a new class of IS3 family. IS family IS4_ssgr_IS231 has 28 copies, IS6 has 13 copies, IS3_ssgr_IS150 has 14 copies, IS1182 and IS110 have two copies, respectively. The pHS18-4 plasmid encodes five copies of IS family IS4_ssgr_IS231 and contains a cry56Aa3 + orf2 gene, the upstream and downstream sequences of cry56Aa3 + orf2 gene have a IS231 insertion sequence, respectively. The pHS18-1 plasmid contains 15 transposase genes and encodes three IS families, of these three families IS200_IS605_ssgr_IS1341 have one copy, IS4_ssgr_IS231 have four copies, and IS200_IS605 have 11 copies. The pHS18-3 plasmid encodes five copies of IS family IS6 while pHS18-5, pHS18-6, pHS18-7, pHS18-8, and pHS18-9 have no IS.

**Table 5:** The varieties and quantities of IS sequences in plasmid pHS18-2.

IS sequence family	Characterization^a^	Start and end position (bp)
IS*200*_IS*605*_ssgr_IS*1341*	0/0/0/1	220,443–222,873
IS*3*_ssgr_IS*3*	0/0/0/1	20,655–22,962
IS*6*	8/5/0/0	5003–2134
		133,763–136,448
		40,062–42,927
		330,134–327,448
		7128–4766
IS*3*_ssgr_IS*150*	8/6/0/0	242,578–245,931
		744–3899
		21,107–23,747
		318,954–316,586
		318,212–315,994
IS*4*_ssgr_IS*231*	0/27/0/1	312,057–308,732
		1–2663
		276,119–278,678
		285,805–283,497
		103,852–107,341
		275,444–278,090
		25,974–23,447
		33,315–30,848
		257,882–255,442
		258,377–256,039
		32,203–29,895
		61,006–63,314
		32,484–30,239
		255,923–253,768
		336,382–337,578
		258,012–255,875
		307,177–309,307
		32,820–30,716
Tn3	0/1/0/0	179,485–183,205
IS*1182*	1/1/0/0	39,248–42,104
		38,601–41,172
IS*110*	2/0/0/0	257,280–254,069

^a^Complete sequence/partial sequence/pseudogenes/unknown sequence.

### Sequence analysis of lysogenic phage of the chromosome

The sequence of HD521 chromosome analysed by PHASTER showed that the HD521 chromosome contains 6 lysogenic phage genome regions (Table 6). Among them, the two sequence regions (sequence 1 and sequence 2) are complete with sequence lengths of 66.3 kbp (2,289,187–2,355,583 bp) and 40.6 kbp (3,817,538–3,858,166 bp) encoding 89 and 29 CDS sequences, respectively. The three sequence regions (sequence 3, sequence 4, and sequence 5) are not complete with lengths of 20.3 kbp (1,167,554–1,187,932 bp), 22.3 kbp (1,994,291–2,016,618 bp), and 23.6. Kbp (3,817,538–3,858,166 bp) encoding 23, 18 and 24 CDS sequences, respectively. Sequence 6 may belong to lysogenic phage, having length of 13.6 kbp (2,014,092–2,027,776 bp) encodes 18 CDS sequences.

**Table 6:** The pro-phage sequences in the chromosome DNA of strain HD521.

Sequence number	Length (kbp)	Number of encoded CDS	Starting position (bp)	Possible prophage	G + C content (%)	Integrity
Sequence 1	66.3	89	2,289,187–2,355,583	PHAGE_Bacill_phBC6A51_NC_004820	37.43	Y
Sequence 2	40.6	29	3,817,538–3,858,166	PHAGE_Geobac_GBSV1_NC_008376	34.59	Y
Sequence 3	20.3	23	1,167,554–1,187,932	PHAGE_Entero_phi92_NC_023693	36.2	N
Sequence 4	22.3	18	1,994,291–2,016,618	PHAGE_Bacill_phBC6A52_NC_004821	34.82	N
Sequence 5	23.6	24	3,817,538–3,858,166	PHAGE_Brevib_Jenst_NC_028805	35.23	N
Sequence 6	13.6	20	2,014,092–2,027,776	PHAGE_Rhizob_vB_RleS_L338C_NC_023502	36.76	Q

Y: complete; N: incomplete; Q: unknown.

Sequence 1 has homology with the prophage sequence of 13 species, 79 of the 89 CDS sequences participate in the coding of phage functional proteins, and 10 CDS sequences encode hypothetical proteins. Sequence 2 has homology with the prophage sequence of 14 species, 26 of the 29 CDS sequences participate in the coding of phage functional proteins, and 3 CDS sequences participate in the coding of hypothetical proteins. Sequence 3 has homology with the prophage sequence of 13 species, 15 of the 23 CDS sequences participate in the coding of phage functional proteins, and 8 CDS sequences participate in the coding of hypothetical proteins. Sequence 4 has homology with the prophage sequence of 7 species, 13 of the 18 CDS sequences participate in the coding of phage functional proteins, and 5 CDS sequences participate in the coding of hypothetical proteins. Sequence 5 has homology with the prophage sequence of 11 species, 17 of the 24 CDS sequences participate in the coding of phage functional proteins, and 7 CDS sequences participate in the coding of hypothetical proteins.

Functional analysis of CDS encoded by prophage sequences that show sequence 1 and sequence 2 contain attachment site Left (attL) and attachment site Right (attR), these sites are specific for the integration of the phage DNA or the excision of the *Bt* HD521 genome. However, the prophage sequence is located between these two attachment sites. Component genes needed by CDS sequence encodes phage integrate or cut with bacterial genome, such as Endolysin, DNA recombination and exonuclease gene, Site-specific recombinase, Exonuclease, and DNA polymerase I encoded by sequence 1. Sequence 2 encodes an Integrase, Resolvase, Site-specific recombinase, Cytokine tail protein, head–tail adaptor, Capsid protein, and Bacterial proteins, etc. Sequence 3 mainly encodes phage-related proteins, such as Tail fiber protein, Calcineurin phosphoesterase, Glycosyltransferase, Collagen triple helix repeat protein, Bacteria encode proteins, and some incomplete phage proteins. Sequence 4 mainly encodes DNA integration, recombination protein, and phage tail protein. Sequence 5 has relatively few CDS that encode functional proteins of phages and does not even contain recombinant related enzymes or phage structural proteins. Sequence 6 has fewer proteins that participate in encoding phages and have only one phage minor tail protein. Similarly, sequences 3, 4, 5, and 6 encode some bacterial-type proteins.

The sequence of HS18-1 chromosome analysed by PHASTER showed HS18-1 chromosome contains 3 lysogenic phage genome regions (Table 7). Among them, sequence 1 is complete and its sequence length is 27.1 kbp (2,506,943–2,534,097 bp) with GC content of 35.77%, and encodes 37 protein sequences. Sequence 2 is incomplete and its length is 20.6 kbp (2,489,098–2,509,752 bp) with GC content of 32.54%, and encodes 24 protein sequences. The length of sequence 3 is 43.2 kbp (1,376,234–1,419,448 bp), its GC content is 34.38%, and encodes 54 protein sequences. Sequence 1 has homology with the prophage sequence of 38 species and its 54.28% of the protein sequence can be aligned with PHAGE_Bacill_phIS3502_NC_019502. Sequence 1 encodes 35 ORFs and a phage-specific attachment site attL and attR, of them 25 ORF sequences encode prophage proteins, such as the transcription regulators of phage ArpU family, site-specific integrases, phage capsid proteins, phage tail assembly proteins, etc.

**Table 7:** The pro-phage sequences in the chromosome DNA of strain HS18-1.

Sequence number	Length (kbp)	Number of encoded CDS	Starting position (bp)	Possible prophage	G + C content (%)	Integrity
Sequence 1	27.1	35	2,506,943–2,534,097	PHAGE_Bacill_phIS3502_NC_019502	35.77	Y
Sequence 2	20.6	23	2,489,098–2,509,752	PHAGE_Bacill_phBC6A52_NC_004821	32.54	N
Sequence 3	43.2	54	1,376,234–1,419,448	PHAGE_Strept_phiARI0131_NC_031941	34.38	Q

Y: complete; N: incomplete; Q: unknown.

Of them 5 ORF sequences encode phage hypothesis proteins, and there are also 5 ORF sequences encode non-phage hypothesis proteins. Sequence 2 has homology with the prophage sequence of 9 species and its 45.45% of the protein sequence can be aligned with PHAGE_Bacill_phBC6A52_NC_004821. Sequence 2 encodes 23 ORFs and a phage-specific attachment site attL and attR, of them 9 ORF sequences encode prophage proteins, such as DNA integration/recombination/insertion protein, DEAD/DEAH box helix protein, Helix-turn-helix protein, Repressor, Replication protein DnaD, Collagen helix repeat protein, etc. Nine ORFs of them encode phage hypothesis proteins, and there are also 4 ORF sequences encode non-phage hypothesis proteins. Sequence 3 has homology with the prophage sequence of 21 species and its 25.92% of the protein sequence can be aligned with PHAGE_Strept_phiARI0131_NC_031941. Sequence 3 encodes 54 ORFs and two phage-specific attachment site attL and attR, of them 24 ORF sequences encode prophage proteins e.g. phage integrin, membrane producing protein, capsid backbone protein, replication initiation protein, RecT recombinase protein, etc. 24 ORF of them encode phage hypothesis proteins, and there are also 8 ORF sequences encode non-phage hypothesis proteins.

## Discussion

By analysing the genome of strain HD521 and HS18-1, we found that these two genomes encode rich virulence factors, such as S-layer protein, enterotoxin, phospholipase, chitinase, and AiiA, etc. They have an important significance for the insecticidal activity and environmental adaptability of *Bt* strains.

Immune Inhibitor A is a metalloproteinase secreted by *Bt*, it is able to degrade antibacterial peptide produced by insects to escape the host's immune system^45,46^. AiiA can hydrolyze AHLs (Acylated Homoserine Lactones) which is bacterial quorum sensing related signaling molecules, its role is to inhibit a variety of bacteria and enhance *Bt*’s competitive advantage in the insect gut^47,48^. Chitinase is a soluble extracellular protein and an insecticidal active substance that can help *Bt* strains to degrade chitin in the peritrophic membrane of insect intestines and make it able to enter in the blood cavity through the perforated intestinal tract to cause insect septicaemia that further enhance the insecticidal protein effect of *Bt* strains^38,49^. Simultaneously, the genome of strain HD521 and HS18-1 also encode abundant plasmids, of which HD521 contains 6 plasmids and HS18-1 contains 9 plasmids. For instance, Plasmid pBTHD521-5 contained in strain HD521, encodes three cry7-like insecticidal crystal protein genes, was cry7Fb3, cry7Ga2 and cry7Da1, respectively.

The plasmid of strain HS18-1 encodes 10 insecticidal crystal protein genes, which are distributed in plasmids pHS18-2, pHS18-4 and pHS18-9, respectively. We revealed that these plasmids carry a large number of transcriptional regulatory factors and genes related to the ABC (ATP-binding cassette) protein transport system. The presence of these genes provides an important theoretical basis for understanding their regulatory mechanisms to positively and negatively regulate companion crystal genes. We also found that the insecticidal genes carried by Bt are almost entirely located on large plasmids, but the whole length of plasmid pHS18-9 is only 7386 bp and encodes a cry54Ba gene in the plasmid of HS18-1.

We found that two genomes have abundant IS including IS200_IS605, IS3, IS4, IS6, IS110 and IS1182, complex transposons Tn3, and junctional transfer system protein, analyzed by horizontal gene transfer of the genomes of HD521 and HS18-1. IS605 belongs to IS200/IS605 family, IS605 is widely distributed in Helicobacter pylori, and its terminal is not an inverted repeat sequence and is a forward repeat. IS605 often forms a complex with IS200. IS200 was originally found in *Salmonella typhimurium*, and its terminal inverted repeat sequence has transposase terminator and block ORF transcription^50^. Moreover, these IS comprised of upstream and downstream sequences of the insecticidal crystal protein, e.g. insertion sequence of IS3 family was comprising downstream sequences of gene cry30Ga1 + orf2, insertion sequence of IS4 and IS6 family were comprising of upstream and downstream sequences of gene cry71Aa1 + orf2 and IS1182 sequence was comprised of downstream sequence of gene cry72Aa1 + orf2. Insertion sequence and transfer system of transposition unit composed by insecticidal crystal protein connected with plasmid is beneficial to horizontal gene transfer of the insecticidal crystal protein gene between the different plasmids of different strains and the different plasmids of same strain, which ultimately plays an important role in the exchange of genetic material and the evolution of population of *B. thuringiensis*.

The IS families and numbers of HD521 and HS18-1 are significantly different in genome distribution, which may be due to the different living environments and population evolution of the two strains. In order to survive and multiply, some strains have enhanced their adaptability to the environment through millions of years of evolution. The insertion sequence has formed a dynamic balance in the adverse and beneficial effects of the host bacteria. The transposition of the insertion sequence mediates genome rearrangement, activates or silences the expression of functional genes, etc., which may cause fatal harm to the host bacteria, and may also enable the host bacteria to acquire new functions, to better adapt to the external environment^51^. For example, there are huge differences in the number of IS4 family insertion sequences in different genomes, which may be mainly related to the living environment and the needs of evolution, but these phenomena show that the insertion sequence plays a very important role in the flexibility and evolution of the genome, rather than a simple "selfish gene"^52^.

Simultaneously, different in the distribution of genome also bring a difference in their functions. Strain HD521 has the characteristics of inhibiting the growth of its hyphae against rice disease-causing bacterium sheath blight AG1 IB (*Rhizoctonia solani* AG1 IB). Simultaneously, the colony of HD521 can produce brown–red pigment, which causes the colony of AG1 IB to appear brown–red, this may be due to the antagonism of multiple microorganisms. It have been reported that *Bacillus thuringiensis* can inhibit a variety of plant diseases caused by filamentous fungi and other plant pathogens. When multiple microorganisms grow together, one kind of microorganism produces one or several specific secondary metabolites in assimilation, which changes its microenvironment, thereby inhibiting or even killing another microorganism^53^.

Strain HS18-1 has high toxicity to lepidopteran and dipteran pests. We analyzed the insertion sequence of the plasmid and found that plasmids containing insecticidal crystal protein genes often contains abundant insertion sequence, and these inserted sequences often form a transposable unit with the insecticidal crystal protein gene. This indicates that the evolution and transfer method of insecticidal crystal protein genes on plasmids of different strains or different plasmids of the same strain is mediated by the insertion sequence.

Plasmid analysis showed that: pHS18-2 is a plasmid, which contains the most insecticidal crystal protein genes, and they are cry30Ga1 + orf2, cry71Aa1 + orf2, cry72Aa1 + orf2, cry70Aa1, cry30Ea1 + orf2, cry69Ab1, cry50Aa1 + orf2, and cry4Cb1. Among them, there is a IS4 family insertion sequence located in the upstream sequence of cry71Aa1 + orf2, IS6 in the downstream and upstream sequence of cry71Aa1 + orf2, cry72Aa1 + orf2, respectively. IS1182 in the downstream sequence of cry72Aa1 + orf2. We analyzed the gene expression of cry71Aa1 and cry72Aa1 and found that these two genes can produce diamond-shaped insecticidal crystal proteins. Insecticidal biological activity testing showed that their crystal protein has good insecticidal activity against the larvae of lepidopteran pests, cotton bollworm, beet armyworm, and diamondback armyworm. The insecticidal crystal protein produced by *Bacillus thuringiensis* is encoded by genes of different sizes, the large-molecular-weight cry proteins are generally encoded by genes above 2 kbp. In this study, cry71Aa1 and cry72Aa1 belong to this type of insecticidal crystal genes. These large-molecular-weight cry genes can produce protein molecules that form independent crystal structures through expression, for example, cryIVD genes can produce irregular hexagonal crystals, and cry8 genes can produce spherical crystals^54,55^.

To date, thousands of *Bt* strains have been identified and isolated but only 24 strains of them found to be fully sequenced The availability and scrutiny of complete genome sequence of strain HD521 and HS18-1 will lay a foundation in *Bt* genome database for further analysis of the generation and regulatory mechanism of cry genes. In summary, the whole genome sequencing and its comparative analysis of both strains (HD521 and HS18-1) will lay out comprehensive perceptions for the genomic diversity and can also be utilized as genomic data support for further strain improvement.
